# 

**Published:** 1903-04

**Authors:** 


					﻿SUBSCRIPTION $1 OO,
ATLANTA PUBLISHED MONTHLY
JOURNAL-RECORD
OF MEDICINE.
Successor to Atlanta Hedical and Surgical Journal, Established 1855,
and Southern fledical Record, Established <870.
Vol. V.	Atlanta, Ga., April, 1903. -------- Nd. 1.
				

